# Patient satisfaction with coronary CT angiography versus invasive coronary angiography: results of a single-center randomized trial

**DOI:** 10.1007/s00330-023-10554-x

**Published:** 2024-02-17

**Authors:** Maria Bosserdt, Viktoria Wieske, Fabian Knebel, Mahmoud M. A. Mohamed, Sarah Feger, Marc Dewey, Eva Schönenberger

**Affiliations:** 1 Departments of Radiology, Charité – Universitätsmedizin Berlin, Charitéplatz 1, 10117 Berlin, Germany; 2 Departments of Cardiology, Charité – Universitätsmedizin Berlin, Charitéplatz 1, 10117 Berlin, Germany; 3 Deutsches Herzzentrum der Charité (DHZC), Charité – Universitätsmedizin Berlin, Berlin, Germany; 4 DZHK (German Centre for Cardiovascular Research), Partner Site Berlin, Berlin, Germany; 5 Departments of Anesthesiology, Charité – Universitätsmedizin Berlin, Charitéplatz 1, 10117 Berlin, Germany

**Keywords:** Computed tomography angiography, Coronary angiography, Coronary artery disease, Patient satisfaction

## Abstract

**Objective:**

Because there is evidence for a clinical benefit of using coronary computed tomography (CT) angiography instead of invasive coronary angiography (ICA) in patients with suspected coronary artery disease (CAD), we ascertained if patient satisfaction could represent an important barrier to implementation of coronary CT in clinical practice.

**Materials and methods:**

A total of 329 patients with suspected CAD and clinical indication for ICA were randomly assigned to undergo either CT or ICA for guiding treatment. Satisfaction for both groups was assessed by patient questionnaire completed twice, ≥24 h after CT or ICA, and at follow-up after a median of 3.7 years. Assessment included preparation, concern, comfort, helplessness, pain, willingness to undergo tests again, overall satisfaction, and preference. Pearson’s chi-square test and Wilcoxon rank-sum test were used.

**Results:**

Overall, 91% of patients undergoing CT (152/167) and 86% undergoing ICA completed assessment (140/162, *p* = 0.19). Patients reported being significantly better prepared for CT, less concerned about the test, and felt less helpless than during ICA (all: *p *< 0.001). Subjective pain (horizontal nonmarked visual analogue scale) was significantly lower for CT (6.9 ± 14.7) than for ICA (17.1 ± 22.7; *p *< 0.001). At follow-up, significantly more patients in the CT group reported very good satisfaction with communication of findings compared with the ICA group (*p *< 0.001) and 92% would recommend the institution to someone referred for the same examination.

**Conclusions:**

Results from our single-center randomized study show very good satisfaction with coronary CT compared to ICA. Thus, superior acceptance of CT should be considered in shared decision-making.

**Clinical relevance statement:**

This evaluation of patient satisfaction in a randomized study shows that patients’ preference is in line with the clinical benefit provided by CT and also suggests to prefer a CT-first strategy in suspected coronary artery disease.

**Key Points:**

• *Subjective pain was significantly lower for coronary CT angiography than for invasive coronary angiography and patients felt better prepared and less concerned about CT.*

• *Patients were overall more satisfied with coronary CT angiography than invasive coronary angiography in a randomized controlled trial.*

• *After a median follow-up of 3.7 years, more patients in the CT group indicated very good satisfaction with the communication of findings and with the examination itself.*

**Supplementary Information:**

The online version contains supplementary material available at 10.1007/s00330-023-10554-x.

## Introduction

Coronary computed tomography (CT) is receiving increasing attention, especially because of higher evidence levels and consequently wider implementation of CT angiography (CTA) for ruling out coronary artery disease (CAD) [1]. CTA is mostly recommended for patients with suspected CAD and low-to-intermediate pretest probability of disease based on results from individual-patient level [2] and study-level meta-analysis [3]. Machine learning of CT datasets is another interesting option that has recently been studied for its potential to better predict future events compared to CAD-RADS scoring alone [4].

Although it is intuitive to assume that patients are more likely to favor noninvasive CT (including a non-contrast acquisition for coronary artery calcium quantification and a post-contrast CT angiography) over the invasive reference standard for diagnosis of CAD—coronary angiography (ICA)—it is imperative to analyze data from the patients’ perspective to thoroughly and critically analyze the possible pros and cons of both testing approaches and patient management strategies. A few studies have investigated the preference, satisfaction, and acceptance of coronary imaging by patients with most of them indicating that CT has relevant advantages [5]. The most recent multicenter CORE-320 study also looked into patient preference and found that coronary CT angiography with stress perfusion was favored over ICA and single-photon emission computed tomography [6]. However, only one study looked at this question in a randomized trial and found that patients randomized to the CT group had a more favorable experience compared to patients randomized to single-photon emission computed tomography [7]. So far, no randomized study investigated patient preference of coronary CT and ICA.

To close this gap in knowledge, we therefore evaluated patient satisfaction with CT and ICA in patients randomly assigned with suspected CAD to CT or ICA in the coronary artery disease management (CAD-Man) study [8] in this prespecified secondary analysis.

## Materials and methods

This prospective randomized study and the study protocol (ClinicalTrials.gov: NCT00844220) were approved by the Charité ethics board and authorized by the German Federal Office for Radiation Protection. The funder of the study, the German Research Foundation, was not involved in any stage of the study design, data acquisition, data analysis, or manuscript preparation. The study’s principal investigator (M.D.) had full control of the data.

### Study design

**Fig. 1:**
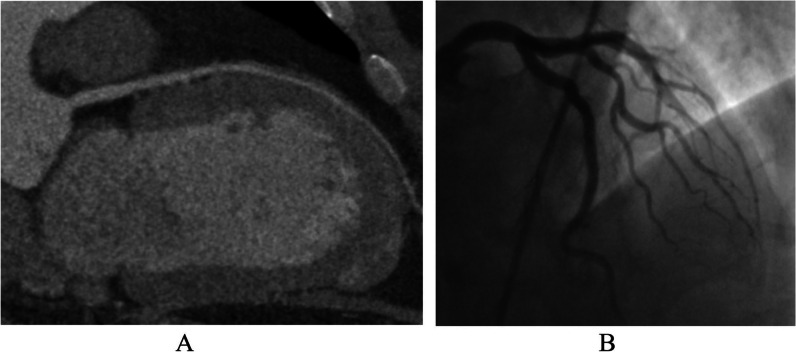
Patient imaging examples. Example of negative coronary CT (**A**) and negative ICA (**B**). **A** Multiplanar reconstruction of the left main and left anterior descending of a patient randomized to coronary CT angiography without pathological findings, CAD was excluded. **B** Patient example of normal invasive coronary angiography showing strong vessels of the left coronary artery and its branches of the left anterior descending and left circumflex artery, CAD was excluded

This study is a prespecified secondary analysis of the CAD-Man trial and is included in the published study protocol [8]. To be eligible for the study and randomization to coronary CT or ICA, patients needed to have suspected CAD and a clinical indication for ICA. In addition, patients had to have a maximum of two of the three criteria for typical angina pectoris (retrosternal chest discomfort, precipitation by exertion, and prompt relief within 30 s to 10 min by rest or nitroglycerine) [8]. The following exclusion criteria were used for the trial: two or more positive tests indicating ischemia, no sinus rhythm, signs of myocardial infarction (persistent ST segment elevation, creatine phosphokinase-MB > 24 U/L, or pulmonary edema due to ischemia), refusal or were incapability of providing informed consent, inability to hold the breath for 5 s, younger than 30 years, or history of or receiving dialysis [8]. The coronary CT procedure included both a non-contrast coronary artery calcium acquisition and a post-contrast CT angiography. No patients had prior coronary CT or ICA. After study enrollment and provision of informed consent, patients were randomly assigned to initial evaluation with either CT or ICA as previously described [8]. If CT was positive for CAD on non-invasive angiographic assessment, patients were then recommended to undergo ICA to confirm the diagnosis before initiation of treatment. Explanations about the imaging tests to the patients prior to their performance were provided by clinical staff. Images were shown during the ICA using on-site monitors by the interventional cardiologists in charge and after the CT procedure using workstations by the radiologists in charge using curved multiplanar reformations and three-dimensional renderings (Fig. 1).

For preference analysis, patients received (1) a questionnaire after the initial examination and (2) follow-up questionnaire. Inclusion criteria for this study were as follows: clinical referral to ICA with suspected CAD based on atypical angina or chest pain. The patients had to be willing to answer the patient preference questionnaire [6] after initial examination and at follow-up. The complete questionnaire used in this study including its follow-up section is available as Online Material A. All study-related actions, such as patient enrollment, randomization, diagnostic procedures, questionnaire evaluation, and statistical analysis, were performed by our group independent of the study sponsor.

### Patient preference questionnaire

Patients were handed the initial management patient preference questionnaire when at least 24 h had passed after CT or ICA. The patient preference questionnaire that is available in eight languages (Chinese, Danish, Dutch, English, German, Indonesian, Japanese, and Portuguese) [6] consisted of a patient satisfaction questionnaire with 8 items regarding their impression of the exam and a comparative questionnaire containing 5 items on patient opinions about the different imaging methods. Patient preparation, concern before the exam, comfort and helplessness during the exam (i.e., perceiving limited control of the situation), satisfaction with the diagnostic pathway, and overall satisfaction were assessed on Likert scale with five levels with the first one being the most positive and the fifth the most negative.

Patients assessed their maximum pain level, resulting, e.g., from puncture or venous access or lying on the table, during or after the procedures, which were performed without sedation, on a 10-cm nonmarked visual analogue scale of 10 cm, which they marked freely. From this, a score of 0 to 100 (measured in mm) indicating no pain to maximum pain was derived. Patients’ willingness to undergo the examination again and the question whether their initial examination had improved or simplified their treatment were assessed with binary items.

Patients also answered a follow-up questionnaire regarding their experience during the initial examination and the subsequent patient management. This instrument was sent to the patients as part of a package of clinical questionnaires at follow-up. In patients who answered the follow-up questionnaires more than once, the last response was included for the current analysis. The follow-up patient preference questionnaire, which was answered by patients at a median of 3.7 years after baseline randomization, again assessed patient satisfaction with the study information, examination preparation, the examination itself, and the reporting of results using a semiquantitative 5-level Likert scale, with the first level being most positive and the fifth being most negative. Information on whether patients were willing to take part in the study again or to have another examination at Charité and whether they would recommend the hospital to other patients was collected as binary items using the follow-up questionnaire.

### Randomization and study procedures

Patients referred for ICA because of suspected CAD were randomized 1:1 to undergo ICA or CT. Randomization procedures were conducted as recently reported [8], as were noninvasive coronary CT angiography and ICA [9].

### Statistical analysis

Differences in outcomes between the two groups were analyzed using Pearson’s chi-square test for nominal or categorical variables and Wilcoxon’s rank-sum test for ordinal Likert scale variables. The unpaired *t*-test was used to identify differences in subjective pain assessed with visual analogue scales. The Benjamini–Hochberg procedure was used to adjust for multiple comparisons. Adjustments for baseline characteristics were not prespecified in the study protocol for this secondary analysis of the CAD-Man trial and were therefore not performed. All statistical analyses were performed with the use of R software, version 4.0.2 (R Foundation for Statistical Computing).

## Results

### Study population

**Fig. 2:**
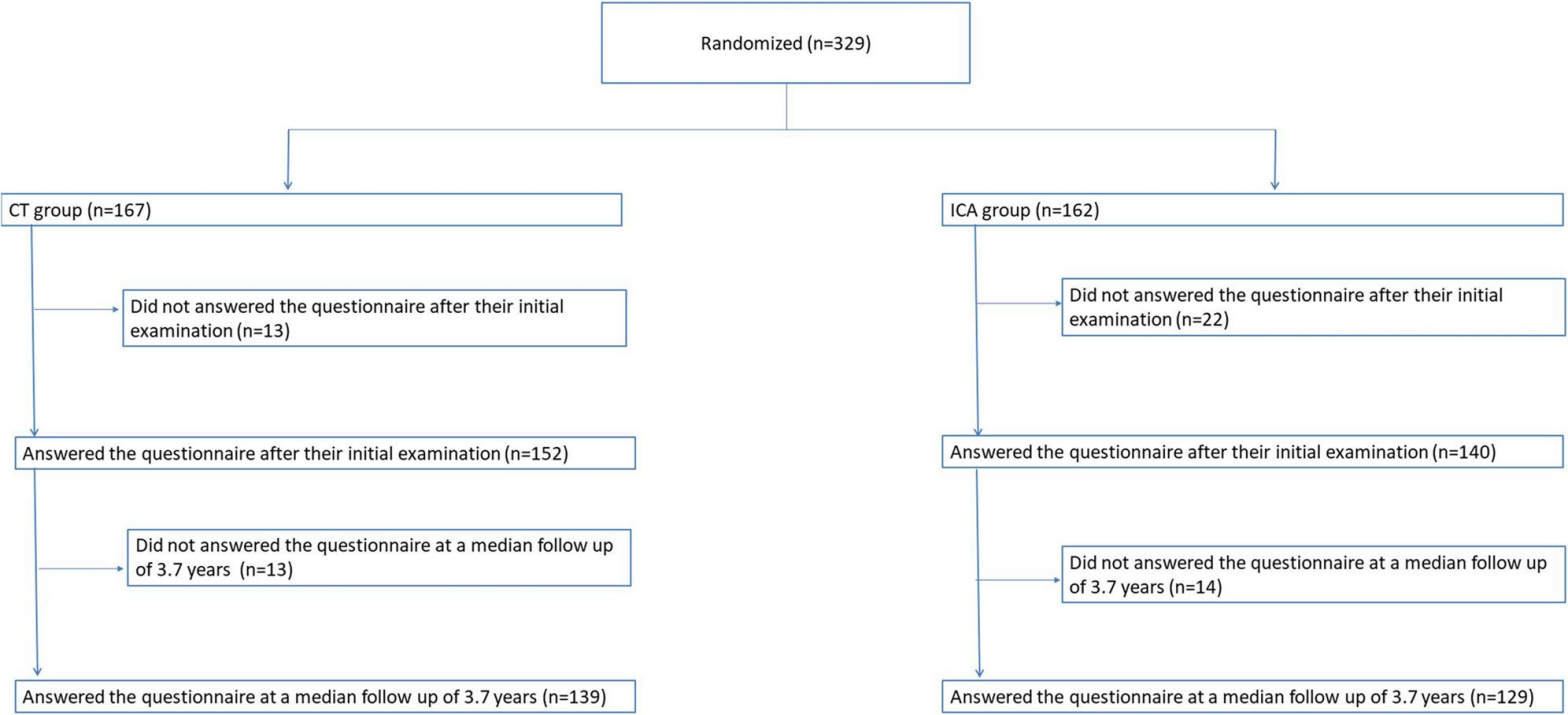
Study flowchart. Consecutive patients with atypical presentation and a clinical indication for ICA based on suspected CAD were assessed for eligibility. For this subanalysis of the patients with the following criteria were available for analysis: performance of initial study test (CT/ICA), completion of patient preference questionnaire at different time points at baseline, after initial study test, and at follow-up

Overall, 292 of the 329 included patients (89%) answered the questionnaire after their initial examination and could be included in this analysis (152/167 (91%) in the CT group and 140/162 (86%) in the ICA group, *p *= 0.19; Fig. 2). Patient baseline and management characteristics including the rat of CAD and revascularizations are summarized in Table 1. No patients had prior coronary CT or ICA. Among the 292 patients who were included with baseline patient preference data, 268 answered at least one of the follow-up preference questionnaires (91%) and were included in the follow-up preference analysis (139/152 in the CT group (92%) and 129/140 in the ICA group (92%; *p *= 0.83)).

**Table 1:** Baseline participant characteristics, findings and management in the two groups

Characteristic	CT (*N* = 152)	ICA (*N* = 140)
Baseline characteristics		
Age (years)	60.3 ± 11.4	60.0 ± 11.1
Female sex, *n (%)*	83 (54.6)	68 (46.6)
Body mass index*	27.4 ± 4.8	27.0 ± 4.5
Hyperlipidemia*, n (%)*	88 (57.9)	74 (52.8)
Arterial hypertension*, n (%)*	100 (65.8)	99 (70.7)
Diabetes mellitus*, n (%)*	13 (8.5)	25 (17.8)
Inpatient, *n* (%)	92 (60.5%)	139 (99.3%)
Outpatient, *n* (%)	60 (39.5%)	1 (0.7%)
Findings and patient management		
Obstructive CAD, *n* (%)	16 (10.5)	22 (15.7)
Revascularization, *n* (%)	14 (9.2)	21 (15)
Staged PCI, *n* (%)	0	1 (0.7%)
Time between randomization and revascularization*	4.5 ± 6.1	6.3 ± 10.0

^*^Continuous measures presented as average with SD

### Pain intensity

**Fig. 3:**
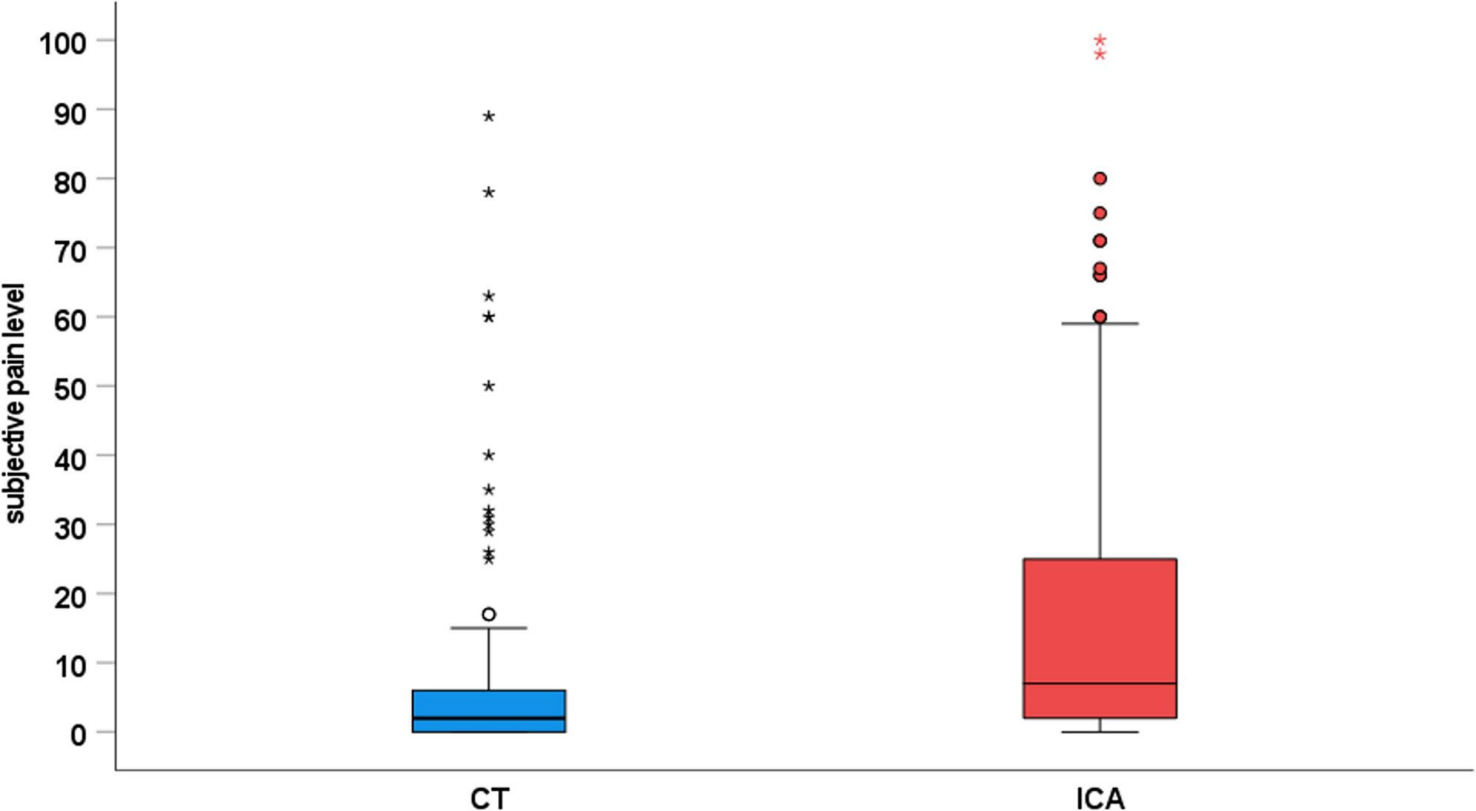
Subjective pain levels reported after examinations in the CT and ICA group. Patients in the CT group had a mean pain level of 6.9 ± 14.7 of 100 on the nonmarked visual analogue scale versus 17.1 ± 22.7 in the ICA group (*p *< 0.001). In the CT group, 62 patients indicated no pain versus 33 patients in the ICA group (*p *= 0.001)

Patients in the CT group had a mean pain level of 6.9 ± 14.7 out of 100 on the nonmarked visual analogue scale while patients in the ICA group had a mean pain level of 17.1 ± 22.7 out of 100 (*p *< 0.001; Fig. 3). In the CT group, 62 patients indicated no pain versus 33 patients in the ICA group (*p *= 0.001). The main pain level was the same between the genders in the ICA group (male 15.4 ± 18.4 vs. female 18.9 ± 26.6; *p *= 0.659) and CT group (male 6.6 ± 16.2 vs. female 7.2 ± 13.4; *p *= 0.62)

### Patient preferences

**Fig. 4:**
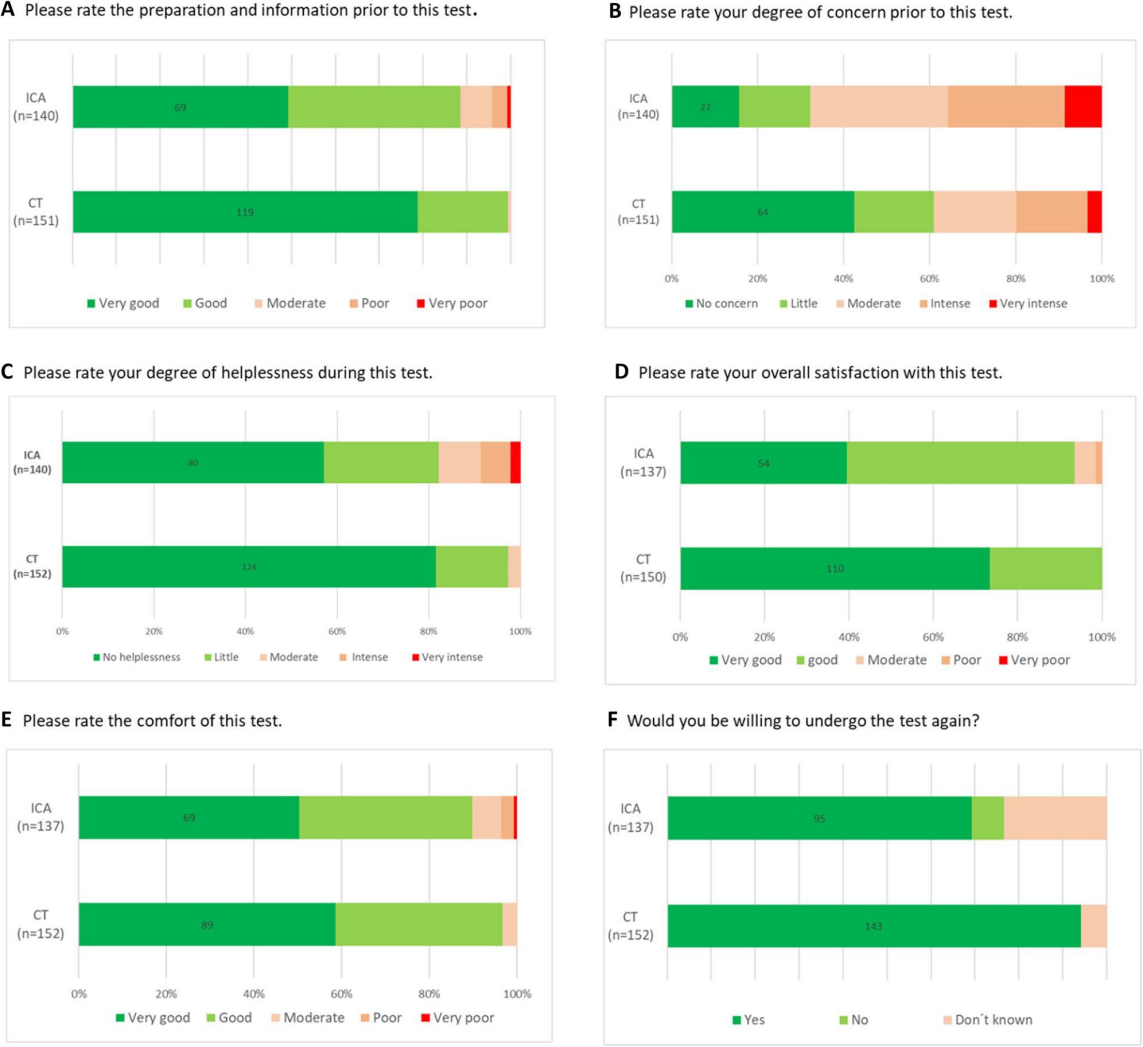
Patient satisfaction with their initial examination in the CT and ICA group. Patients reported that they were significantly better prepared for CT (*p *< 0.001; **A**), less concerned about CT (*p *< 0.001; **B**), had a lesser degree of helplessness (*p *< 0.001; **C**), and higher overall satisfaction (*p *< 0.001; **D**), while comfort showed no tendency to be greater (*p *= 0.74; **E**) compared to ICA. In the CT group, more patients would be willing to undergo the test again compared to the ICA group (*p *< 0.001; **F**)

Patients reported that they were significantly better prepared for CT (median (Mdn) = 1 vs. Mdn = 2, Mann-Withney *U* (*U*) = 7226, *z*-score (*z*) = − 5.55, *p *< 0.001; Fig. 4A; Supplementary Fig. E2 A), were less concerned about CT (Mdn = 2 vs. Mdn = 3, *U *= 6994, *z *= − 5.14 *p *< 0.001; Fig. 4B; Supplementary Fig. E2 B), had a lesser degree of helplessness (Mdn = 1 vs. Mdn = 1, *U *= 7786, *z *= - 4.91 *p *< 0.001; Fig. 4C; Supplementary Fig. E2 C), and had higher overall satisfaction (Mdn = 1 vs. Mdn = 2, *U *= 6610, *z *= − 6.02, *p *< 0.001; Fig. 4D) compared to ICA. The rate of comfort was the same in the two groups (Mdn = 1 vs. Mdn = 1, *U *= 9732, *z *= − 1.42, *p *= 0.074 Fig. 4E; Supplementary Fig. E2 E). In the CT group, more patients would be willing to undergo the test again compared to the ICA group (*p *< 0.001; Fig. 4F; Supplementary Fig. E2 F). Importantly, no differences were found for other parameters of patient preference between genders in both group (Table 2).

**Table 2:** Patient acceptance stratified by gender

Characteristic*	CT		*U*	*z*	*p*	ICA		*U*	*z*	*p*
	Female (*n *= 83) Mdn	Male (*n *= 69) Mdn				Female (*n *= 68) Mdn	Male (*n *= 72) Mdn			
Please rate the preparation and information prior to this test	1	1	2635	− 0.99	0.324	1	1	2406	− 0.19	0.848
Please rate your degree of concern prior to this test	2	2	2386	− 1.74	0.082	3	3	2288	− 0.69	0.492
Please rate the comfort of this test.	1	1	2811	− 0.22	0.822	1	1	2368	− 0.36	0.715
Please rate your degree of helplessness during this test	1	1	2503	− 1.98	0.048	1	1	2303	− 0.67	0.498
Please rate your overall satisfaction with this test.	1	1	2695	− 0.42	0.675	2	2	2183	− 0.77	0.444

^*^Numbers in the columns CT/ICA are referring to the scale 1–5 according to the patient’s rating as follows: Very good (1), good (2), moderate (3), poor (4), very poor (5). See also Supplementary Material for original patient satisfaction questionnaire

*Mdn* median, *U* Mann-Withney *U*, *Z z*-score

### Open-text responses

In the open-text responses, 130 patients (45%) gave a reason for concern before the examination (50 of 152 patients in the CT group (33%), 80 of 140 in the ICA group (57%)). The three most common reasons for concern in the ICA group were fear of complications during the procedure (45 patients), mainly related to presence of unspecified fear of complications (31 patients) and injury caused by invasive procedure (5 patients), fear of examination findings (15 patients), and fear of an unknown examination (13 patients). The three most common reasons for concern in the CT group were fear of examination findings (23 patients), fear of complications (16 patients), mainly related to contrast agent (10 patients), and concerns about comfort during the examination (5 patients). A detailed overview of all open-text responses by category can be found in the Supplementary Material (Supplementary Fig. E1).

### Follow-up questionnaire results

**Fig. 5:**
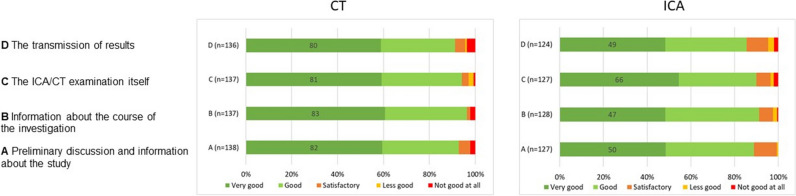
Patient satisfaction at long-term follow-up. After a median follow-up of 3.7 (CI 95% 3.6 – 4.0) years, patients in the CT group were more satisfied with information about the study (*p *= .002; **A**), with information about the course of the investigation (*p *< 0.001; **B**), with transmission of results (*p *< 0.001; **D**), compared to the ICA group. No difference was found in regards to the examination itself between both groups (*p *= 0.19; **C**)

At a median follow-up of 3.7 years (CI 95% 3.6 – 4.0), patients in the CT group were more satisfied with information about the study (Mdn = 1 vs. Mdn = 2, *U *= 7016, *z *= 0.002, *p *= 0.002; Fig. 5A), with communication of findings (Mdn = 1 vs. Mdn = 2, *U *= 6745, *z *= − 1.31, *p* = 0.002; Fig. 5D), and with information about the course of the examination compared to the ICA group (Mdn = 1 vs. Mdn = 2, *U *= 6566, *z *= − 3.96 *p *< 0.001; Fig. 5B). There was no statistically significant difference between the two groups regarding the examination itself (*U *= 7985, *z *= − 1.31, *p* = 0.19; Fig. 5C). If given the choice, most of the patients would have participated in the study again, with patients in the CT group being more likely to participate again (107/135 or 80% in the CT vs. 78/128 or 60% in the ICA group; *p* = 0.001). The majority of patients from both groups would undergo their examination at Charité again (CT 125/137; 91%; ICA 103/128; 80%; *p *= 0.001) and 92% would recommend Charité to someone referred for the same examination (CT 130/136 95%; ICA 112/126; 88% ICA; *p* = 0.046).

## Discussion

In the presence of evidence for a clinical benefit of using coronary CT instead of ICA in patients with suspected CAD from the single-center CAD-Man [8] and the multicenter DISCHARGE trial [10, 11], we ascertained if patient satisfaction could represent an important barrier to implementation of coronary CT in clinical practice. Without randomized studies, one cannot conclude on the long-term satisfaction of a CT-directed versus and ICA-directed treatment strategy. The principal findings of this randomized trial are as follows: (1) subjective pain was significantly lower in patients who underwent coronary CT angiography compared to those who underwent invasive coronary angiography; (2) patients felt better prepared and less concerned about the CT examination; and (3) there was significantly greater satisfaction with the communication of findings in the CT-first group, which also persisted at follow-up after 3.7 years.

Previous studies suggest that patients with suspected coronary disease might prefer noninvasive imaging to invasive angiography for diagnostic workup. In an intraindividual comparison, Schönenberger et al used the same patient satisfaction questionnaire and found that coronary CT angiography was less painful than ICA and more comfortable than coronary magnetic resonance angiography [5]. Feger et al also used this questionnaire and showed that all noninvasive cardiac imaging tests were well accepted by patients with CT being the preferred examination, compared with perfusion magnetic resonance imaging [12]. In a multicenter investigation of coronary imaging combined with stress perfusion using a version of the present questionnaire expanded for the functional tests, Minhas et al found coronary CT angiography with stress perfusion to be favored over ICA and single-photon emission computed tomography [6]. This was corroborated in the only randomized trial of coronary CT angiography and single-photon emission computed tomography investigating patient preference, in which Levsky et al showed a more favorable experience in the CT group using a different questionnaire [7]. There are no randomized studies available on the patient satisfaction with CT and ICA. This gap in knowledge is important because direct comparisons of satisfaction in patients who had both CT and ICA were done before and are limited by “contamination” bias; i.e., patients may mix up satisfaction from both tests or procedure-related complications from one test may actually influence the satisfaction with the other. Therefore, the most important clinical implication of the current randomized trial analysis is that patient preference for coronary CT should be considered when discussing with patients their individual preference for testing with ICA or CT, and the evidence-based information provided by the present study regarding short- and long-term satisfaction with coronary CT and ICA should be included in the shared decision-making discussion between health care providers and patients.

Patient preference is also important in other fields of imaging, and, besides cardiac imaging, has been most widely studied for colonic imaging. Florie et al, for instance, showed that MR colonography without extensive cleansing was preferred to colonoscopy both immediately after examinations and 5 weeks later [13]. In another analysis of this study by van Gelder et al, CT colonography was also favored over colonoscopy, yet this preference for CT decreased over time [14]. For CT colonography, Lefere et al found that fecal tagging offers a well-tolerated preparation while improving visibility of polyps [15]. Zalis et al compared patients who received fecal tagging preparations for CT colonography with control groups and found improved patient comfort scores using fecal tagging with best results achieved for a hyperosmotic laxative and a nonionic contrast agent [16]. The SIGGAR multicenter investigators conclude that CT colonography has superior patient acceptability compared with colonoscopy in the short term, but colonoscopy offers some benefits to patients that become apparent after longer-term follow-up [17]. In our study, the general preference for CT compared with invasive coronary angiography did not change over time.

In general, patient preference is going to become an important indicator for the likelihood of widespread clinical implementation and success of novel radiological imaging methods. Patient satisfaction with imaging as well as communication of findings, which persisted over long-term follow-up in our analysis, will also contribute to patient-centered care in radiology [18]. It is unknown if the use of structured compared with unstructured reporting in radiology and specifically using CAD radiology reporting (CAD-RADS) [19] will further improve the preference for CT by patients or if direct communication of results to patients by radiologists is more relevant. Improving patient experience in radiology is important and can be accomplished by using a multifaceted quality improvement initiative [20]. Patient satisfaction constitutes a core component of the shift from volume- to value-based radiology [21], which also includes better patient satisfaction, outcomes, and overall system savings, as also summarized by Rubin et al for the International Society of Strategic Studies in Radiology [22]. Providing appropriate information about procedures to patients before testing was shown to be important [23] and was similar between the two randomization groups in our study.

There are several mechanisms that could have contributed to the greater satisfaction and lower pain connected with CT compared with ICA in our patients. First, CT is a noninvasive imaging test and usually does not cause pain, unless there is inadvertent contrast agent extravasation. More than one-third of patients in the CT group did not report any pain (59 of 151), and pain levels in the CT group were generally low and mostly related to back pain because of lying flat. Second, coronary CT angiography might be perceived by patients as more novel and the three-dimensional renderings as more exciting than fluoroscopy images during ICA. This may have been perceived as a possible subjective advantage of CT with an impact on patient preference even though the patient information about this randomized study highlighted that CT was the investigational test in this comparative effectiveness study. Third, the majority of patients in this study did not require further invasive procedures including coronary revascularization, and satisfaction with clinical pathways in the two randomization groups was greater for ICA in patients who needed revascularization. Thus, in study populations with a higher risk of coronary disease, patients might perceive CT as being less advantageous, highlighting that CT should be primarily used in patients with low-to-intermediate pretest probability of CAD [2] and that further clinical studies are needed about satisfaction in patients with high clinical CAD likelihood.

Our study has relevant limitations. It was conducted at a single site, albeit in a randomized fashion. The majority of patients filled the preference questionnaire yet completing it was, albeit insignificantly, more likely in the CT (90%) compared to the ICA group (86%). It is therefore possible that few patients from the ICA group with very high or low satisfaction did not complete the questionnaire. The questionnaire, which is available in eight languages, was not formally validated using independent data from other studies or for response bias. Moreover, a common problem with surveys of patients is that respondents might try to please the observers or perceive new test as higher technology which may influence results. Follow-up data were also available, yet only in 93% of patients who could be included at baseline. Another possible bias that cannot be excluded arises from the study team having been possibly engaged to a greater extend in the process in patients in the CT group. While preference was evaluated also in subgroups of patients undergoing versus not requiring coronary revascularization, the study population was too small to compare preference and satisfaction in terms of different approaches to ICA (femoral versus radial arterial access) and different approaches to revascularization (interventional versus surgical). The same limitation for subgroup analyses applies to outpatient ICA procedures which were rarely performed in the current cohort. This needs to be investigated in future studies including more patients. Additionally, coronary CT angiography has some test specific limitations that may limit its overall utility in some patients, such as in those with arrhythmias, obesity, inability to hold the breath, or very severe claustrophobia. In terms of validity, several additional variables that are relevant to patients have not been assessed. These include the time required for the investigations. Since this study was completed, the European and American cardiology societies have proposed guidelines that favor CT as a first-line investigation in stable chest pain and suspected CAD [24, 25]. Valid determination of what patients want and what matters most to them was not included in these guidelines and such complexity is likely to be beyond the capacity of most patients to provide meaningful comments in a survey. From a study reporting perspective, it is important to note as a limitation that there are currently no reporting guidelines and checklists to follow for patient satisfaction studies.

In summary, in patients with low to moderate risk of suspected CAD, patient satisfaction was in favor of coronary CT angiography compared to ICA indicating the potential of CT, also reflected in emerging guidelines, to become a widely accepted and possible primary clinical imaging test in this patient group.

### Supplementary Information

Below is the link to the electronic supplementary material.

Supplementary file1 (PDF 275 KB)

## Abbreviations

CAD: Coronary artery disease
CAD-Man: Coronary Artery Disease Management Study
CT: Computed tomography
CTA: Computed tomography angiography
ICA: Invasive coronary angiography
Mdn: Median
*U*: Mann-Whitney *U*
*Z*: *z*-score

## Declarations

### Guarantor

The scientific guarantor of this publication is Prof. Dr. Marc Dewey.

### Statistics and biometry

MB and MM have significant statistical expertise.

### Study subjects or cohorts overlap

The study cohort was reported in the CAD-Man main study report (https://www.bmj.com/content/355/bmj.i5441).

### Methodology

• Prospective

• Randomized controlled trial

• Performed at one institution
