# Supplementary material for: Patient satisfaction with coronary CT angiography versus invasive coronary angiography: results of a single-center randomized trial
Source: Eur Radiol. 2024 Feb 17;34(9):5644–53. doi: 10.1007/s00330-023-10554-x (PMC11364721; doi:10.1007/s00330-023-10554-x)
Supplement: Supplementary file 1 — Supplementary file1 (PDF 275 KB) [file 330_2023_10554_MOESM1_ESM.pdf]

Patient satisfaction with coronary CT angiography versus invasive coronary angiography: results of a single-center randomized trial

Electronic Supplementary Material (ESM)

Eur Radiol (2023) Bosserdt M, Wieske V, Knebel F et al

Electronic Supplementary Material A

Patient Satisfaction Questionnaire Patient Acrostic:

CT (Computed Tomography)

1. Please rate the preparation and information prior to this test.

   Very good ❍ Good ❍ Moderate ❍ Poor ❍ Very poor ❍

2. Please rate your degree of concern prior to this test.

   No concern ❍ Little ❍ Moderate ❍ Intense ❍ Very intense ❍

   In case you were concerned: **Why** were you concerned? ……………………………..

3. Please rate the comfort of this test.

   Very good ❍ Good ❍ Moderate ❍ Poor ❍ Very poor ❍

4. Please rate your degree of helplessness during this test.

   No helplessness ❍ Little ❍ Moderate ❍ Intense ❍ Very intense ❍

5. Please rate your pain during and immediately after this test on the following scale.

   No pain |————————————————| *Maximum pain*

6. In Your opinion, were there any complications during or after the test?

   Yes ❍ No ❍ If yes, what kind were these ❍

7. Would you be willing to undergo the CT test again?

   Yes ❍ No ❍ Don’t know ❍

8. Please rate your overall satisfaction with this test.

   Very good ❍ Good ❍ Moderate ❍ Poor ❍ Very poor ❍

Catheterization

1. Please rate the preparation and information prior to this test.

   Very good ❍ Good ❍ Moderate ❍ Poor ❍ Very poor ❍

2. Please rate your degree of concern prior to this test.

   No concern ❍ Little ❍ Moderate ❍ Intense ❍ Very intense ❍

   In case you were concerned: **Why** were you concerned? ……………………………..

3. Please rate the comfort of this test.

   Very good ❍ Good ❍ Moderate ❍ Poor ❍ Very poor ❍

4. Please rate your degree of helplessness during this test.

   No helplessness ❍ Little ❍ Moderate ❍ Intense ❍ Very intense ❍

5. Please rate your pain during and after this test on the following scale.

   No pain |————————————————| *Maximum pain*

6. In Your opinion, were there any complications during or after the test?

Yes ❍ No ❍ If yes, what kind were these ❍

7. Would you be willing to undergo this test again?

Yes ❍ No ❍ Don't know ❍

8. Please rate your overall satisfaction with this test.

Very good ❍ Good ❍ Moderate ❍ Poor ❍ Very poor ❍

Summary

1. Which test would you prefer for future imaging for suspected coronary artery disease?

CT ❍ Catheterization ❍

2. Please describe other important thoughts about the three tests on the following lines and explicitly name the advantages and disadvantages of CT, and Catheterization using the table below.

……………………………………………………………………………………………………

……………………………………………………………………………………………………

……………………………………………………………………………………………………

	Advantages	Disadvantages
CT		
Catheterization		

3. How do you rate the overall satisfaction with the treatment path randomized to (CT or Catheterization)?

Very good ❍ Good ❍ Moderate ❍ Poor ❍ Very poor ❍

4. If you could have selected the treatment path, which one would it have been?

CT ❍ Catheterization ❍ not sure ❍, Please describe why you would decide like this or why you are not sure

…………………………………………………………………………………………………

…………………………………………………………………………………………………

…………………………………………………………………………………………………

Patient Satisfaction Questionnaire at Follow-up Patient Acrostic (CT exam):

1. Were you satisfied with the study in the following points:

Please check the relevant box

Rating: 1 = very good, 2 = good, 3 = satisfactory, 4 = less good, 5 = not good at all

Preliminary discussion and information about the study	**1❍**	**2❍**	**3❍**	**4❍**	**5❍**
Information about the course of the investigation	**1❍**	**2❍**	**3❍**	**4❍**	**5❍**
The CT examination itself	**1❍**	**2❍**	**3❍**	**4❍**	**5❍**
The transmission of results	**1❍**	**2❍**	**3❍**	**4❍**	**5❍**

2. From the current perspective, would you participate in a study like this again?

   ❍ **yes** ❍ **no**

3. Would you have another CT scan at the Charité?

   ❍ **yes** ❍ **no**

4. Would you recommend a CT scan at the Charité to a friend?

   ❍ **yes** ❍ **no**

5. Do you have any comments, requests or criticism?

   ❍ **yes, in fact**:____

   ____

   ❍ **no**

Patient Satisfaction Questionnaire at Follow-up Patient Acrostic (ICA exam):

1. Were you satisfied with the study in the following points:

Please check the relevant box

Rating: 1 = very good, 2 = good, 3 = satisfactory, 4 = less good, 5 = not good at all

Preliminary discussion and information about the study	**1❍**	**2❍**	**3❍**	**4❍**	**5❍**
Information about the course of the investigation	**1❍**	**2❍**	**3❍**	**4❍**	**5❍**
Catheterization examination itself	**1❍**	**2❍**	**3❍**	**4❍**	**5❍**
The transmission of results / copy of results	**1❍**	**2❍**	**3❍**	**4❍**	**5❍**

2. From the current perspective, would you participate in a study like this again?

   ❍ **yes** ❍ **no**

3. Would you have another catheterization at the Charité?

   ❍ **yes** ❍ **no**

4. Would you recommend a CT catheterization at the Charité to a friend?

   ❍ **yes** ❍ **no**

5. Do you have any comments, requests or criticism?

   ❍ **yes, in fact**:____

   ____

   ❍ **no**

Supplemental Figures

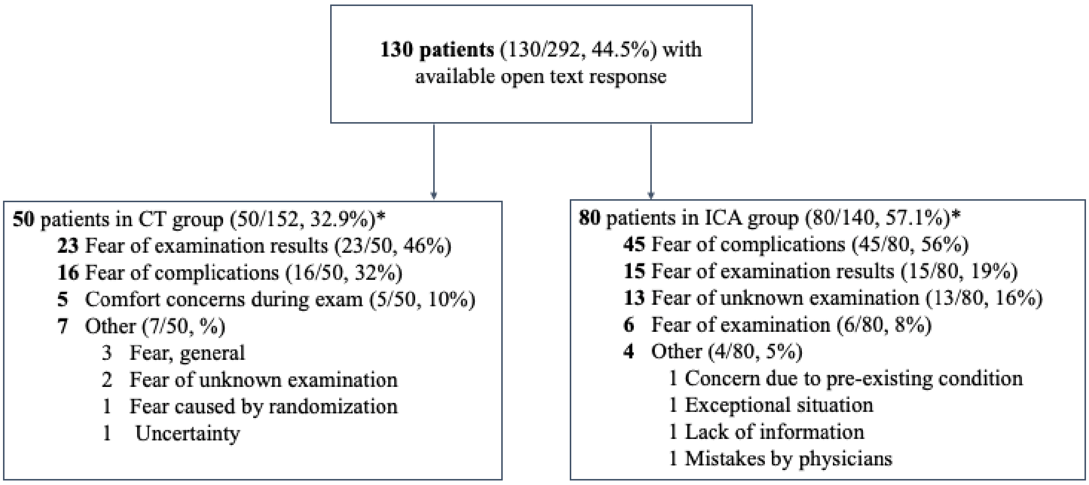

Fig E1: Detailed list of all text responses by category for patients in the CT and ICA group, respectively.

CT, computed tomography; ICA, invasive coronary angiography

* Multiple responses per patient possible, thus, the sum of numbers in bold (parent category) for CT (n=51) and ICA (n=83) group is unequal to the number of patients available for open text responses (CT: 50 patients; ICA: 80 patients).

Figure E2: Patient satisfaction with examinations in the CT and ICA group.

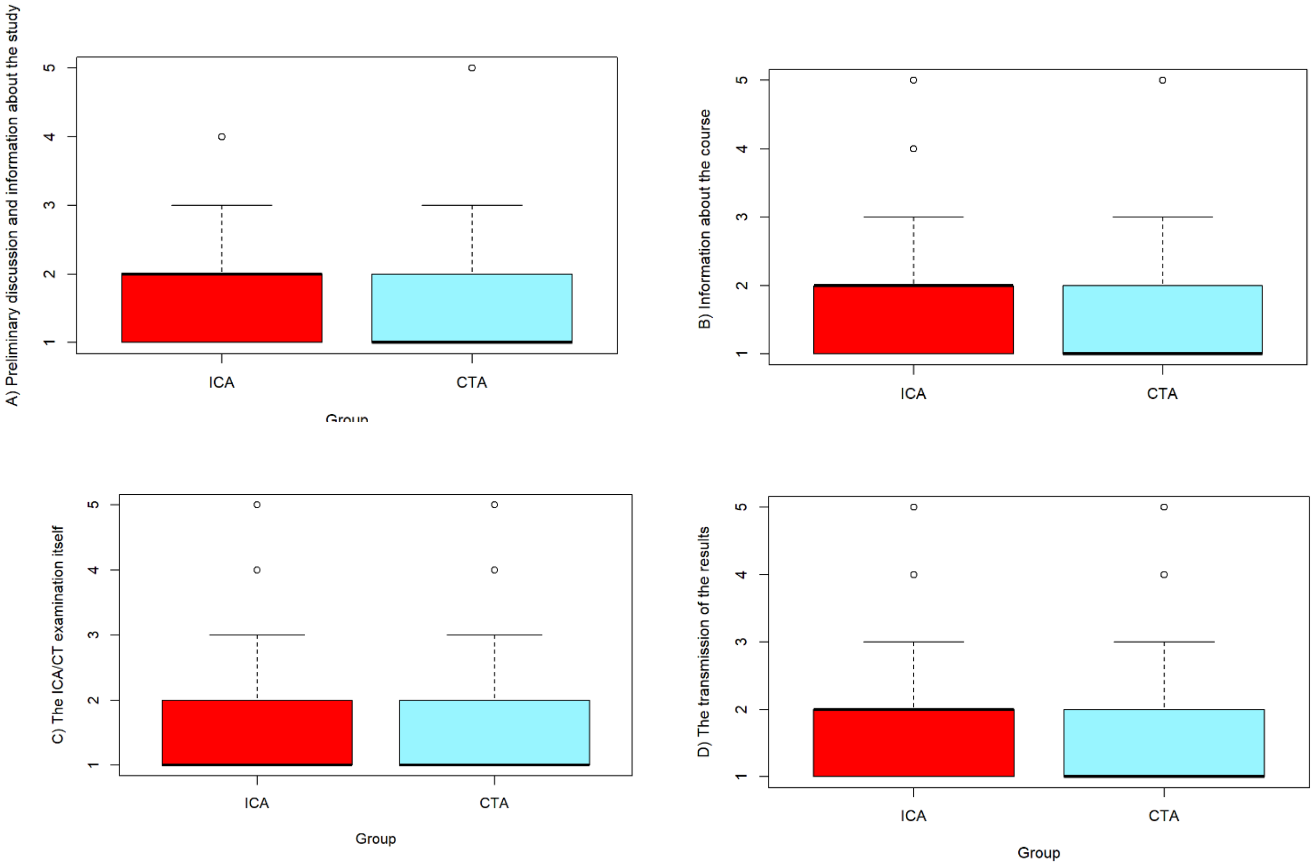

Patients reported that they were significantly better prepared for CT (p <0.001; **Fig E2 A**), less concerned about CT (p <0.001; **Fig E2 B**), had a lesser degree of helplessness (p<0.001; **Fig E2 C**), and higher overall satisfaction (p<0.001; **Fig E2 D**), while no difference in regard to tendency for greater comfort (p=0.074; **Fig E2 E**) compared to ICA. In the CT group more patients would be willing to undergo the test again compared to the ICA group (p<0.001; **Fig E2 F**).
